# Human *Schistosoma haematobium* Antifecundity Immunity Is Dependent on Transmission Intensity and Associated With Immunoglobulin G1 to Worm-Derived Antigens

**DOI:** 10.1093/infdis/jiu374

**Published:** 2014-07-07

**Authors:** Shona Wilson, Frances M. Jones, Govert J. van Dam, Paul L. A. M. Corstjens, Gilles Riveau, Colin M. Fitzsimmons, Moussa Sacko, Birgitte J. Vennervald, David W. Dunne

**Affiliations:** 1 Department of Pathology, University of Cambridge, United Kingdom; 2 Department of Parasitology; 3 Department of Molecular Cell Biology, Leiden University Medical Center, The Netherlands; 4 CIIL, Inserm U1019, Pasteur Institute, Lille, France; 5 Institut National de Recherche en Santé Publique, Bamako, Mali; 6 Centre for Health Research and Development, Faculty of Life Sciences, University of Copenhagen, Frederiksberg, Denmark

**Keywords:** *Schistosoma haematobium*, human, immunity, transmission, fecundity

## Abstract

**Background:**

Immunity that reduces worm fecundity and, in turn, reduces morbidity is proposed for *Schistosoma haematobium*, a parasite of major public health importance. Mathematical models of epidemiological trends suggest that antifecundity immunity is dependent on antibody responses to adult-worm-derived antigen.

**Methods:**

For a Malian cohort (age, 5–29 years) residing in high-transmission fishing villages or a moderate-transmission village, worm fecundity was assessed using the ratio of urinary egg excretion to levels of circulating anodic antigen, a *Schistosoma*-specific antigen that is steadily secreted by adult worms. Fecundity was modeled against host age, infection transmission intensity, and antibody responses specific to soluble worm antigen (SWA), tegument allergen-like 1, and 28-kDa glutathione-S-transferase.

**Results:**

Worm fecundity declined steadily until a host age of 11 years. Among children, host age and transmission were negatively associated with worm fecundity. A significant interaction term between host age and transmission indicates that antifecundity immunity develops earlier in high-transmission areas. SWA immunoglobulin G1 (IgG1) levels explained the effect of transmission on antifecundity immunity.

**Conclusion:**

Antifecundity immunity, which is likely to be protective against severe morbidity, develops rapidly during childhood. Antifecundity immunity is associated with SWA-IgG1, with higher infection transmission increasing this response at an earlier age, leading to earlier development of antifecundity immunity.

Parasitic worms of the genus *Schistosoma* cause debilitating chronic infections affecting an estimated 240 million people [1]. *Schistosoma haematobium* is the most prevalent form, with World Health Organization estimates, updated from those previously reported [2], indicating that it currently accounts for 112 million infections and 24 million cases of bladder morbidity [3]. Long-term consequences of infection include hydronephrosis, which can lead to kidney failure [4] and squamous cell bladder carcinoma [5]. *S. haematobium* also causes female genital schistosomiasis, facilitating human immunodeficiency virus transmission [6].

Studies examining immunity to schistosomes have mainly concentrated on anti-infection immunity, an immunoglobulin E (IgE)–mediated process [7] that develops through intermittent exposure to previously cryptic adult-worm-derived antigens upon worm death, with *S. haematobium* estimated to live for 3.4 years in the human host [8]. Increased exposure to these cryptic antigens leads to the expansion of schistosome-specific IgE antibody targets, including members of the tegument-allergen-like (TAL) family [9], ultimately including ones that are shared or cross-reactive between the adult worm and the skin-invading schistosomule [10]. A peak shift in the development of immunity is proposed, as individuals in areas of higher infection transmission are exposed to higher levels of cryptic antigens at an earlier age [11].

However, as morbidity is caused by immunopathogenic responses to schistosome eggs trapped within tissues, with one of the greatest contributing factors being the number of eggs deposited [4, 12–14], immunity that protects against morbidity, rather than against infection, could target worm fecundity. A pattern-oriented mathematical model derived from field-based trends in urinary egg counts and schistosome-specific antibody patterns predicts that, for *S. haematobium*, the main protective immunity is antifecundity immunity and that, similar to anti-infection immunity, this immunity is dependent on antigen exposure upon death of adult worms [15].

Experimental support for antifecundity immunity comes from studies on *Schistosoma bovis*, a parasite in the same clade as *S. haematobium*, in which cattle previously chronically exposed to *S. bovis* have reduced egg excretion per adult worm pair upon infection challenge, compared with naive cattle [16]. This antifecundity immunity can be serum transferred, indicating an antibody-mediated process [17]. One proposed target is 28-kDa glutathione-S-transferase (GST), vaccination with which reduces worm fecundity in patas monkeys experimentally infected with *S. haematobium* [18] and in *S. bovis*–infected cattle [19].

In humans, assessment of worm burden has to be indirect. The most appropriate tool for this is measurement of circulating anodic antigen (CAA), a *Schistosoma*-specific, gut-derived glycoprotein [20, 21] steadily secreted by the worms during the regurgitation that occurs because of their blind-ended guts [22]. In a *S. haematobium–*endemic population, urinary egg excretion to circulating CAA level in adults is less than that in children, indicative of an antifecundity effect in adults [23]. Here, we assess further the relationship between egg excretion and CAA in *S. haematobium* infection, examining for the first time the impact of differing transmission intensities on worm fecundity. We also examine the association of GST and specific antibody responses to adult-worm-derived antigen, including anti-TAL1 responses, a proxy of worm death, with worm fecundity.

## MATERIALS AND METHODS

### Study Area and Population

The study took place in Segou Region, Mali. Two villages with high-intensity transmission, Kaladangan and Guenidaga (n = 140), are neighboring fishing settlements on the Niger River. A third village, with moderate-intensity transmission, Kalabougou (population, 153 individuals), is located on a tributary of the main river and has a population with a diverse range of occupations, including fishing, farming, and pottery. The analysis was conducted for an age-restricted (5–29 years) subcohort of a larger cohort, aged 3–40 years (population, 500 individuals), randomly selected from the combined village populations for participation in a multidisciplinary study. Three urine samples were collected from each individual before treatment, and 10 mL of each was filtered to determine *S. haematobium* egg counts. The arithmetic mean of the 3 counts was used for analysis. All participants were treated with either a single dose of praziquantel (40 mg/kg) or 2 doses administered 2 weeks apart. No difference in treatment efficacy was detected for the 2 treatment regimens [24]. Pre-treatment *S. mansoni* was detected at a low prevalence (*n* = 31, 15%) and intensity (median 48 eggs per gramme faeces, in those with detectable eggs) by Kato–Katz [24] of a single stool sample. No eggs of gut helminths were detected.

Oral informed consent was given by adult participants and by parents/guardians of participating children. Because of cultural reasons and low literacy rates in villages, the Malian Ministry of Health deem oral consent acceptable. The study was approved by the Ethical Review Committee of the National Institute for Research in Public Health (decision 0002/INRSP/DAP/SP- 2005).

### Antigen Production

Soluble recombinant ShTAL1 was produced by amplifying the coding sequence with amended restriction sites from the pTrcHis vector containing ShTAL1 that had been generated previously [26] and ligating it into expression vector pGEX-4T-3 (GE Healthcare, Little Chalfont, United Kingdom) between BamHI and EcoRI restriction sites. ShTAL1 was then expressed, isolated, and purified as described previously for SmTAL1 [9]. Sh28GST was produced as previously described [27]. SWA was extracted as previously described [28] from worms recovered from mice by portal perfusion.

### Serology

Before treatment, 5 mL of blood was collected by venipuncture into ethylenediaminetetraacetic acid, and plasma was harvested by centrifugation. Plasma samples were treated with 0.3% tributyl phosphate/1% Tween 80 (both from Sigma, Poole, United Kingdom) to inactivate encapsulated viruses.

SWA-IgG1, -IgG4, -IgE, and -IgA; Sh28GST-IgG1, -IgG3, and -IgA; and ShTAL1-IgG1, -IgG4, and -IgE were measured in duplicate by enzyme-linked immunosorbent assay (ELISA). Plates containing 384 wells were coated with 8 µg/mL SWA antigen, 20 µg/mL Sh28GST or 2.4 µg/mL ShTAL1. For SWA specific antibodies, plasma was diluted 1:400 for IgG1, 1:200 for IgG4, 1:60 for IgA, and 1:20 for IgE. For Sh28GST-specific antibodies, plasma was diluted 1:200 for IgG1, 1:100 for IgG3, and 1:100 for IgA. For ShTAL1-specific antibodies, plasma was diluted 1:100 for IgG1, 1:200 for IgG4, and 1:20 for IgE. Biotinylated anti-human IgG1 clone G17-1, IgG4 clone G17-4, IgA clone G20-359, and IgE clone G7-26 (all from BD Pharmingen, San Diego, CA) were used to detect bound antibody. IgG3 was detected using biotinylated anti-human IgG3 clone HP6047 (Zymed, San Francisco, CA). Assays were developed using OPD substrate (Sigma). Antibody concentrations were extrapolated from standard curves of purified human IgG1, IgG4, and IgA (all from Sigma) and purified human IgG3 and myeloma IgE (both from Calbiochem, Darmstadt, Germany). Because of the low levels detected, ShTAL1 responses were defined as responder/nonresponder with a cutoff of mean + 3 SDs of the response measured in a panel of 26 non–schistosome-infected European/North American plasma samples.

A 40-µL aliquot of plasma was sent to Leiden University Medical Centre for CAA measurement as previously described [29]. CAA concentrations in samples were calculated from a standard curve and expressed as pg/mL plasma.

### Statistical Analysis

Before treatment, 17 individuals were negative by egg count and CAA and were removed from the analysis because they were presumed to be uninfected. The response variable, worm fecundity, was examined by linear regression. Worm fecundity was calculated as follows: [(number of eggs excreted per 10 mL of urine +1)/(pg of CAA per mL of plasma + 1)]. Findings were log transformed prior to analysis. Dependence of worm fecundity on host age was examined for improved model fit, using piecewise regression. The fit of the piecewise regression model with the lowest residual standard error was compared with a nonpiecewise regression model by analysis of variance.

For individuals aged <11 years, the dependence of worm fecundity on host antibody levels and transmission intensity (high transmission, 56 individuals; moderate transmission, 75 individuals), including interaction terms between host age and transmission, were explored. Models were reduced by stepwise removal of nonsignificant predictors, and the results are expressed as the geometric mean (GM) ratio.

The presence of *S. mansoni* adults will contribute to CAA levels, reducing the apparent *S. haematobium* egg to adult worm ratio. For cultural reasons, the provision of stool samples was low, resulting in a number of missing data points (70 of 276). Multiple imputation by chained equations, using predictive mean matching and 25 imputations, was used to estimate these missing values (Supplementary Figure 1). Predictors in the imputation were age, transmission intensity, sex, ln(caa + 1), ln(Sh eggs + 1), ln(SWA-IgG1), ln(SWA-IgG4), ln(SWA-IgE) and ln(SWA-IgA), ln(Sh28GST-IgG1), ln(Sh28GST-IgG3), ln(Sh28GST-IgA), detectable ShTAL1-IgG1, and IgG4 and IgE responses, with complete data sets available for all. In models of worm fecundity for individuals aged <11 years, the reported statistics are the pooled fits from models adjusted for the 25 imputations of *S. mansoni* infection intensity.

The association between SWA-IgG1–specific responses and host age and transmission intensity were analyzed by linear regression analysis.

## RESULTS

### Association Between Host Age, Transmission Intensity, and Worm Fecundity

**Figure 1.**
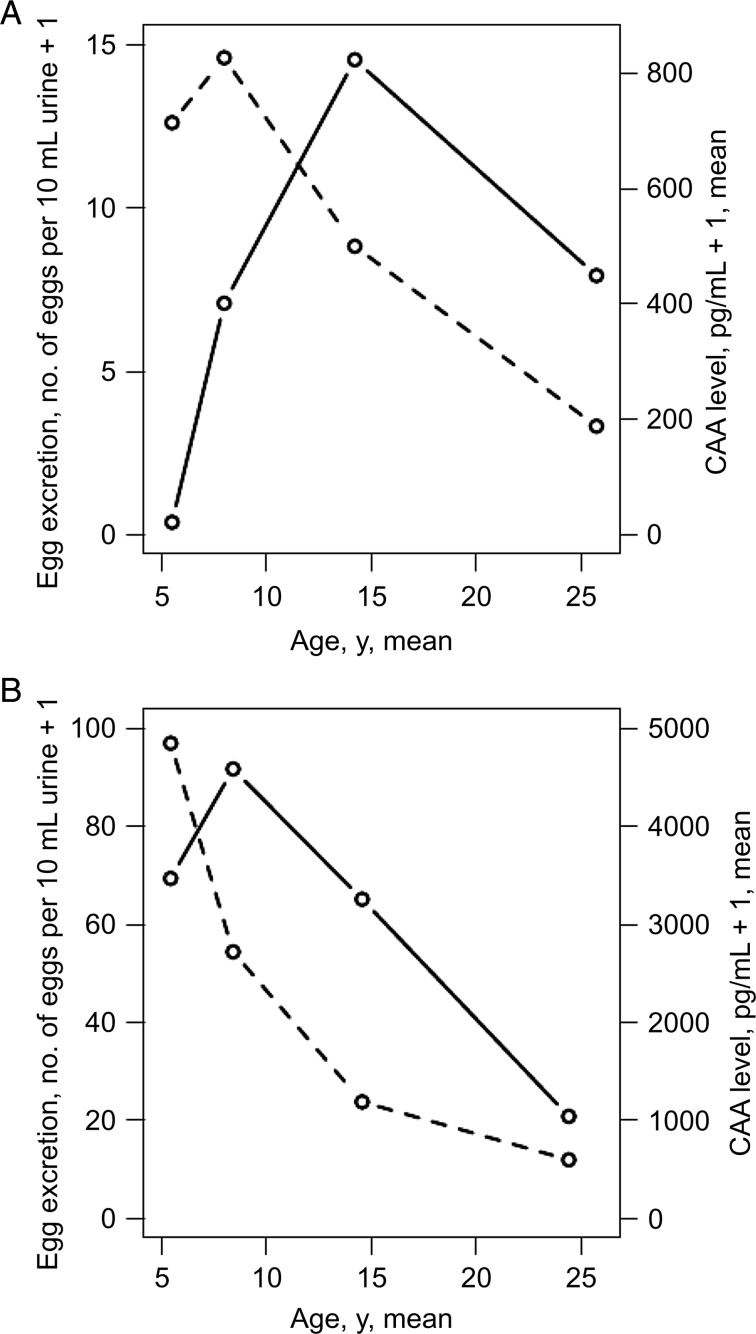
Age-based curves of egg excretion and worm burden in moderate-transmission and high-transmission villages. Shown are geometric mean egg counts (per 10 mL of urine + 1) (dotted line) and the geometric mean picograms of circulating anodic antigen (CAA; per milliliter of plasma + 1; solid line) for the moderate-transmission village (*A*) and the high-transmission villages (*B*).

The GM egg counts and worm burdens, stratified by age, for the participating villages are shown in Figure 1. For both transmission intensities, worm burden followed characteristic age-infection intensity curves, in which infection intensity increases during childhood and then declines. The peak in CAA levels occurred after the peak in urinary egg excretion. The peak in CAA levels and egg excretion was at an earlier age in the high-transmission villages.

**Figure 2.**
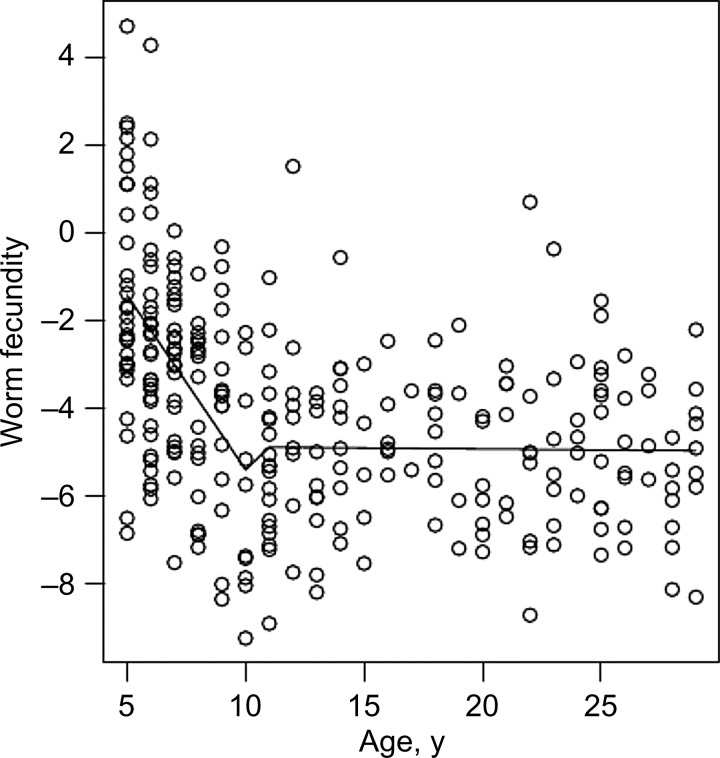
Worm fecundity with host age. Scatterplot of worm fecundity, defined as [(number of eggs excreted per 10 mL of urine +1)/(picograms of circulating anodic antigen per milliliter of plasma + 1)], versus host age and the fitted regression lines for individuals aged <11 years and ≥11 years.

When worm fecundity was expressed as a ratio of egg excretion to CAA levels, a nonlinear relationship was observed between host age and worm fecundity (Figure 2). Worm fecundity steadily declined until 10–12 years of age, after which it was flat in relation to host age. Piecewise regressions with breaks at host ages of 7–16 years were explored. Breaking the regression at 11 years returned the smallest residual standard error (Supplementary Figure 2). This piecewise regression model had a significantly better fit than a nonpiecewise linear regression model (*F* = 27.277; *P* < .001). In the piecewise regression model, for individuals aged ≥11 years, there was no significant relationship between age and worm fecundity (β = 0.005; *P* = .875), but individuals <11 years of age had both a significantly greater *y*-axis intercept (β = 7.374; *P* < .001) and a significantly different slope of the regression line (β = −0.789; *P* < .001), compared with those aged ≥11 years (Figure 2). The smallest residual standard error remained at 11 years of age, and the same significant associations between host age and worm fecundity remained after piecewise regression analysis was adjusted for imputed *S. mansoni* infection intensities (Supplementary Table 1).

**Figure 3.**
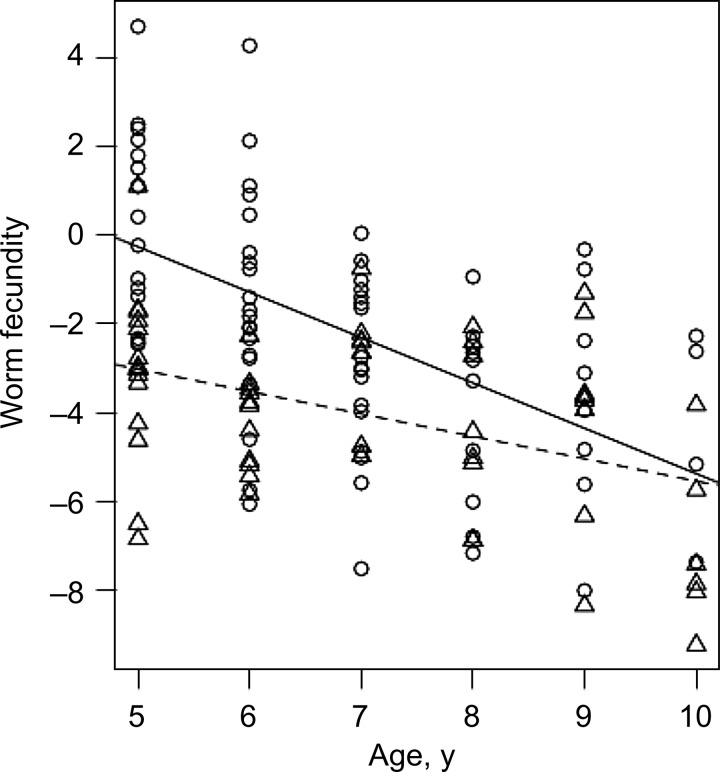
Worm fecundity with host age and transmission. Scatterplot of worm fecundity, defined as [(number of eggs excreted per 10 mL of urine +1)/(picograms of circulating anodic antigen per milliliter of plasma + 1)], versus host age for children aged <11 years. Circles indicate residence in the moderate-transmission mixed-occupation village, and the solid line indicates the fitted regression line for these children. Triangles indicate residence in the high-transmission fishing villages, and the dotted line indicates the fitted regression line for these children.

The association between host age and worm fecundity was explored further in individuals <11 years of age, taking into account the sex of the host, the differing transmission intensities, and the interaction between host age and transmission. There was a significant negative relationship between host age and worm fecundity (GM ratio, 0.359; 95% confidence interval [CI], .26–.50; *P* < .001). Lower worm fecundity occurred in children residing in the high-transmission villages (GM ratio, 0.005; 95% CI, 2 × 10^−4^–.15; *P* = .002). An interaction term between host age and transmission was also significant (GM ratio, 1.677; 95% CI, 1.05–2.69; *P* = .032), reflecting the lower gradient of the host age/worm-fecundity slope in the higher-transmission villages. This was caused by the lower intercept on the *y*-axis for children in these higher-transmission villages (Figure 3). Host sex was not a significant predictor of worm fecundity and was removed from the model (GM ratio, 1.38; 95% CI, .64–2.94; *P* = .405).

### Associations Between Schistosome-Specific Antibody Responses and Worm Fecundity

IgG1, IgG4, IgE, and IgA responses to SWA were individually entered into univariant linear regression models of worm fecundity for those aged <11 years. All 4 antibody responses were significantly negatively associated with worm fecundity, but IgG1 responses explained the greatest variance in worm fecundity, with the lowest residual standard error (Table 1). SWA-IgG1 levels were entered into a multivariant model of worm fecundity for individuals aged <11 years that contained age, transmission intensity and the age-transmission interaction term. SWA-IgG1 levels remained a significant negative predictor of worm fecundity in this model (GM ratio, 0.70; 95% CI, .59–.82; *P* < .001), but neither the interaction term between age and transmission (GM ratio, 1.52; 95% CI, .94–2.44; *P* = .087) nor transmission intensity itself (GM ratio, 0.50; 95% CI, .15–1.50; *P* = .247) remained significant after the addition of SWA-IgG1 and were removed from the model. Both age (GM ratio, 1.28; 95% CI, 1.06–1.55; *P* = .010) and transmission intensity (GM ratio, 68.19; 95% CI, 37.38–124.37; *P* < .001) were significant predictors of SWA-IgG1. Therefore, the transmission-driven increase in SWA-IgG1 levels explains the earlier decrease in worm fecundity observed in the high transmission villages. Age remained a significant negative predictor of worm fecundity (GM ratio, 0.51; 95% CI, .40–.65; *P* < .001), indicating that other age-dependent mediators of antifecundity immunity exist.

**Table 1.** Association of Worm-Specific Antibody Responses With Worm Fecundity

Antibody	GM Ratio (95% CI)	Adjusted R^2^	SE
IgG1	0.652 (.56–.76)^a^	0.193	2.37
IgG4	0.723 (.64–.81)^a^	0.189	2.38
IgE	0.674 (.54–.85)^a^	0.076	2.54
IgA	0.554 (.32–.96)^b^	0.027	2.60

Shown are the relationship strengths (geometric mean [GM] ratios), the explained variance (adjusted R^2^), and residual standard error (SE) of univariant linear regression models of worm fecundity, calculated as [(number of eggs excreted per 10 mL of urine +1)/(picograms of circulating anodic antigen per milliliter of plasma + 1)], for 4 antibody classes/subclasses specific to soluble worm antigen.

Abbreviations: CI, confidence interval; IgA, immunoglobulin A; IgE, immunoglobulin E; IgG, immunoglobulin G.

^a^ *P* < .001

^b^ *P* < .05.

When SWA-IgG1 was replaced by SWA-IgG4, the antibody isotype with the next best fit in univariant analysis of worm fecundity, the interaction term between age and transmission was no longer significant (GM ratio, 1.49; 95% CI, .93–2.40; *P* = .097), but transmission itself remained significant (GM ratio, 0.30; 95% CI, .12–.73; *P* = .009).

Sh28GST-IgG1 and Sh28GST-IgG3 levels were not significantly associated with worm fecundity in models adjusted for age, transmission intensity, and the interaction term between the 2 (GM ratio, 0.96 [95% CI, .72–1.29; *P* = .726] and 0.90 [95% CI, .75–1.08; *P* = .246]), respectively). Sh28GST-IgA was a significant negative predictor of worm fecundity in a comparable model (GM ratio, 0.84; 95% CI, .71–1.00; *P* = .047). However, when added to a model adjusted for age and SWA-IgG1 level, the negative association between Sh28GST-IgA and worm fecundity was no longer significant (GM ratio, 0.86; 95% CI, .73–1.02; *P* = .087).

ShTAL1-IgG1 responses were not significantly associated with worm fecundity in models adjusted for age, transmission intensity, and the interaction term between the 2 (GM ratio, 0.59; 95% CI, .27–1.27; *P* = .174), but ShTAL1-IgG4 and ShTAL1-IgE responses were significant negative predictors of worm fecundity in comparable models (GM ratio, 0.33 [95% CI, .13–.88; *P* = .027] and 0.34 [95% CI, .12–.97; *P* = .045], respectively). When added to a model adjusted for age and SWA-IgG1 level, the negative associations between worm fecundity and ShTAL1-IgG4 and ShTAL1-IgE responses were no longer significant (GM ratio, 0.38 [95% CI, .14–1.03; *P* = .058) and 0.46 [95% CI, .16–1.30; *P* = .140], respectively).

## DISCUSSION

Because adult schistosome worms live in the bloodstream of the host, it is not possible to directly measure the worm burden in humans. Here, we measured the worm burden indirectly by using a sensitive up-converting phosphor technology-based lateral flow CAA assay [29, 30]. The secretion of CAA by adult worms is not influenced by the immune state of the host [31], so age-dependent immune responses will not confound CAA measurements. The observed earlier peak in *S. haematobium* egg excretion in comparison to CAA levels is therefore indicative that, in this population, egg excretion is not in balance with worm burden. Although some alteration in the host's ability to transfer eggs across the bladder wall into the bladder lumen cannot be fully discounted, the observed significant associations with host age, village, and antibody responses to worm-derived antigens suggests that a transmission-dependent humoral immune response directed against the adult worms is reducing their fecundity.

*S. mansoni*, the other African schistosome of public health importance, was present in the cohort, but among those who provided a stool sample, infection was at a low prevalence and intensity. Although there were fewer stool than urine samples provided by the study population, the distribution of *S. mansoni* infection intensity for those who provided stool samples closely mirrored that of those who did not, via the multiply imputed *S. mansoni* infection intensities. The addition of *S. mansoni* infection intensity to models did not influence the significant associations seen between age, transmission intensity, and antibody responses and the ratio of *S. haematobium* egg excretion to CAA (worm fecundity).

In a previous study, antifecundity immunity was proposed to exist for *S. haematobium*, developing by 15 years of age, but not for *S. mansoni* [23]*,* for which deviations away from a linear relationship between CAA and egg count have been attributed to crowding effects reducing the fitness of the adult worms [32]. It is not possible to completely rule out crowding effects in this study because of the observed high infection intensities. However, worm fecundity decreased between hosts aged 5 and 10 years in the high-transmission villages, ages at which CAA levels were higher than those among hosts aged 20–29 years, so the development of antifecundity immunity, rather than crowding effects, is the more likely explanation for the decrease in worm fecundity.

Changes in host hormone levels at puberty could possibly explain the observed reduction in egg excretion from the age of 11 years in this study and from the age of 15 years reported in a previous study [23]. However, the significant age-interaction term in the model for 5–10-year-old subjects suggests that, in the high-transmission villages, antifecundity immunity starts to develop prior to puberty, with some children 5 years old having developed a degree of antifecundity immunity.

*S. haematobium* is phylogenetically within a clade of schistosomes infecting ungulates and other primates, and it is so closely related to some members that hybridization occurs [33, 34]. It may therefore be more appropriate to address the question of antifecundity immunity with data obtained from experimental infection of ungulates with other clade members than human *S. mansoni* infections. In *S. bovis* experimental infections, antifecundity immunity can be transferred via serum [17], and vaccination with Sb28GST leads to a decrease in fecundity [19], a finding reproduced in Sh28GST vaccination/*S. haematobium* infection challenge experiments of patas monkeys [18]. In the current study, Sh28GST-IgG1 and -IgG3 levels were not associated with worm fecundity, and the negative association initially observed for Sh28GST-IgA was no longer significant upon addition of SWA-IgG1 responses to the model. This does not necessarily negate the argument that anti-Sh28GST responses have a role in antifecundity immunity. Sh28GST is strongly immunogenic, giving rise to high IgG1 and IgG3 responses in phase 1 vaccination trials in naive European adult males [27]. IgG3 responses are also high in chronically infected males, while IgA responses are high in chronically infected females [35]. However, in vaccination experiments of cattle with *S. bovis* and the closely related *S. matteei,* the ability of sera to inhibit GST function is not related to antibody levels [36], indicating that maturation of GST-inhibiting antibody responses is also required.

A pattern-oriented mathematical model predicts that antibody to antigens exposed upon worm death mediate antifecundity immunity [15]. Here, we measured responses to ShTAL1 as a proxy of exposure to cryptic worm antigens. ShTAL1 is a homologue of a *S. mansoni* cryptic tegument-associated protein, and the pattern of the antibody responses to ShTAL1 are similar to those of SmTAL1, with IgG4 and IgE levels increasing with age and after treatment [26]. Additionally, life-stage-expression analysis has shown that ShTAL1, like its *S. mansoni* homologue, is predominantly expressed in the adult worm (H. Dickenson and C. M. Fitzsimmons, unpublished data). ShTAL1-IgG4 and ShTAL1-IgE responses were negatively associated with worm fecundity, after adjustment for age and transmission, indicating that antigens exposed upon worm death may give rise to antibody responses that mediate antifecundity immunity. However, after adjustment for SWA-IgG1, these relationships were no longer significant.

SWA is an extract from adult worms that includes numerous antigens, many of which are cryptic during the life of the worm, with some found on the surface or excreted/secreted and others shared/cross-reactive with other life stages of the parasite. Those on the surface or excreted/secreted and those shared by the eggs (because eggs die in the tissues) will be continuously exposed to the host. It was levels of SWA-IgG1 that were explanatory of the observed transmission intensity dependence of antifecundity immunity, indicating that this isotype may play a predominant role. IgG1 is raised against cryptic antigens, as indicted by a boost in levels after treatment induced worm death [37], but it is also raised in response to antigens to which the host is regularly exposed [38, 39]. The association between SWA-IgG1 and worm fecundity does not therefore clarify whether the targets of this immunity are cryptic antigens. However, SWA-IgG1, because of its explanatory role in the association observed between anti-TAL1 responses and worm fecundity, gives weight to the argument that antifecundity immunity arises from exposure to worm-derived cryptic antigens.

To conclude, a reduction in worm fecundity was observed during childhood and developed earlier in higher-transmission villages. The IgG1 responses to worm antigen were explanatory of this transmission dependence, implicating this antibody isotype in antifecundity immunity. The specific target of this response remains to be clarified, but the results add impetus to research into candidates for a potential *S. haematobium* antifecundity/morbidity vaccine, which would improve the quality of life for millions of individuals.

## Supplementary Data

Supplementary materials are available at *The Journal of Infectious Diseases* online (http://jid.oxfordjournals.org). Supplementary materials consist of data provided by the author that are published to benefit the reader. The posted materials are not copyedited. The contents of all supplementary data are the sole responsibility of the authors. Questions or messages regarding errors should be addressed to the author.

Supplementary Data
