# Supplementary material for: Human Schistosoma haematobium Antifecundity Immunity Is Dependent on Transmission Intensity and Associated With Immunoglobulin G1 to Worm-Derived Antigens
Source: J Infect Dis. 2014 Jul 7;210(12):2009–16. doi: 10.1093/infdis/jiu374 (PMC4241947; doi:10.1093/infdis/jiu374)
Supplement: Supplementary Data [file supp_210_12_2009__index.html]

Human Schistosoma haematobium anti-fecundity immunity is dependent on transmission intensity and is associated with IgG1 to worm-derived antigens — Human Schistosoma haematobium Antifecundity Immunity Is Dependent on Transmission Intensity and Associated With Immunoglobulin G1 to Worm-Derived Antigens — Human Schistosoma haematobium Antifecundity Immunity Is Dependent on Transmission Intensity and Associated With Immunoglobulin G1 to Worm-Derived Antigens — Supplementary Data 

# Human *Schistosoma haematobium* Antifecundity Immunity Is Dependent on Transmission Intensity and Associated With Immunoglobulin G1 to Worm-Derived Antigens

## Supplementary Data

Supplementary Data

**Files in this Data Supplement:**

- Supplementary Data - Docx file
