# Supplementary material for: Human Schistosoma haematobium Antifecundity Immunity Is Dependent on Transmission Intensity and Associated With Immunoglobulin G1 to Worm-Derived Antigens
Source: J Infect Dis. 2014 Jul 7;210(12):2009–16. doi: 10.1093/infdis/jiu374 (PMC4241947; doi:10.1093/infdis/jiu374)
Supplement: Supplementary Data [file supp_jiu374_jiu374supp.docx]

**Supplementary Material**

**Fig. S1. Density distributions for observed and imputed values of *S. mansoni* infection intensity.**

All imputations show plausible distributions, with 24/25 showing a close fit with the density distributions of the observed data. Only imputation 6 shows a deviation, with a higher number of imputed values being the equivalent of no eggs detected.

**Fig. S2. The standard error of residuals from piecewise regression models**

Piecewise regressions were broken at host-ages 7-16yrs, the best model fit occurs when the regression is broken at 11yrs of age.

**Table S1. Piecewise regression analysis of adult worm fecundity adjusted for imputed *S. mansoni* infection intensities**

|  | Age (>=11-yrs) | | Intercept (<11yrs) | | Slope (<11yrs) | |
| --- | --- | --- | --- | --- | --- | --- |
|  | β (S.E.) | p-value | β (S.E.) | p-value | β (S.E.) | p-value |
| Imp 1 | -0.011 (0.03) | 0.699 | 7.182 (1.00) | <0.001 | -0.780 (0.12) | <0.001 |
| Imp 2 | -0.005 (0.03) | 0.855 | 7.294 (1.00) | <0.001 | -0.780 (0.12) | <0.001 |
| Imp 3 | -0.005 (0.03) | 0.867 | 7.321 (0.99) | <0.001 | -0.783 (0.12) | <0.001 |
| Imp 4 | -0.010 (0.03) | 0.735 | 7.393 (0.98) | <0.001 | -0.799 (0.12) | <0.001 |
| Imp 5 | -0.007 (0.03) | 0.810 | 7.301 (0.99) | <0.001 | -0.786 (0.12) | <0.001 |
| Imp 6 | -0.006 (0.03) | 0.827 | 7.290 (1.00) | <0.001 | -0.783 (0.12) | <0.001 |
| Imp 7 | -0.009 (0.03) | 0.761 | 7.240 (0.99) | <0.001 | -0.780 (0.12) | <0.001 |
| Imp 8 | -0.007 (0.03) | 0.824 | 7.325 (0.99) | <0.001 | -0.787 (0.12) | <0.001 |
| Imp 9 | -0.007 (0.03) | 0.820 | 7.246 (0.99) | <0.001 | -0.775 (0.12) | <0.001 |
| Imp 10 | -0.007 (0.03) | 0.813 | 7.339 (0.99) | <0.001 | -0.789 (0.12) | <0.001 |
| Imp 11 | -0.006 (0.03) | 0.823 | 7.306 (0.99) | <0.001 | -0.784 (0.12) | <0.001 |
| Imp 12 | -0.007 (0.03) | 0.811 | 7.263 (1.00) | <0.001 | -0.778 (0.12) | <0.001 |
| Imp 13 | -0.012 (0.03) | 0.675 | 7.175 (0.98) | <0.001 | -0.775 (0.12) | <0.001 |
| Imp 14 | -0.006 (0.03) | 0.825 | 7.461 (0.99) | <0.001 | -0.803 (0.12) | <0.001 |
| Imp 15 | -0.004 (0.03) | 0.899 | 7.370 (1.00) | <0.001 | -0.790 (0.12) | <0.001 |
| Imp 16 | -0.007 (0.03) | 0.802 | 7.341 (0.99) | <0.001 | -0.787 (0.12) | <0.001 |
| Imp 17 | -0.006 (0.03) | 0.824 | 7.431 (0.98) | <0.001 | -0.799 (0.12) | <0.001 |
| Imp 18 | -0.005 (0.03) | 0.857 | 7.361 (1.00) | <0.001 | -0.791 (0.12) | <0.001 |
| Imp 19 | -0.005 (0.03) | 0.873 | 7.376 (0.99) | <0.001 | -0.794 (0.12) | <0.001 |
| Imp 20 | -0.007 (0.03) | 0.815 | 7.260 (0.99) | <0.001 | -0.777 (0.12) | <0.001 |
| Imp 21 | -0.008 (0.03) | 0.779 | 7.236 (0.99) | <0.001 | -0.777 (0.12) | <0.001 |
| Imp 22 | -0.009 (0.03) | 0.758 | 7.320 (0.99) | <0.001 | -0.788 (0.12) | <0.001 |
| Imp 23 | -0.006 (0.03) | 0.841 | 7.318 (1.00) | <0.001 | -0.784 (0.12) | <0.001 |
| Imp 24 | -0.008 (0.03) | 0.770 | 7.312 (0.99) | <0.001 | -0.790 (0.12) | <0.001 |
| Imp 25 | -0.008 (0.03) | 0.785 | 7.300 (0.99) | <0.001 | -0.785 (0.12) | <0.001 |

Shown are β-coefficients, standard errors (S.E.) and p-values for individuals >=11yrs-old, and the difference in the intercept and slope for individuals <11yrs-old in piecewise regression analysis adjusted for 25 imputations of *S. mansoni* infection intensity.
